# Chinese Expert Consensus on the Clinical Application of Finerenone in Geriatric Comorbidities: Geriatrics Branch of Chinese Medical Association, Cardiovascular Group of Geriatrics Branch of Chinese Medical Association

**DOI:** 10.1002/agm2.70103

**Published:** 2026-08-20

**Authors:** Xiaoming Wang, Cuntai Zhang

**Affiliations:** ^1^ Department of Geriatrics, Xijing Hospital Air Force Medical University Xi'an China; ^2^ Department of Geriatrics, Tongji Hospital, Tongji Medical College Huazhong University of Science and Technology Wuhan China

**Keywords:** Finerenone, geriatric comorbidities, mineralocorticoid receptor antagonist

## Abstract

Dysregulation of the renin‐angiotensin‐aldosterone system (RAAS) constitutes a pivotal factor in the onset and progression of various geriatric diseases, including cardiovascular diseases, diabetes, and chronic kidney disease (CKD), all closely linked to adverse cardiorenal outcomes in older patients. Excessive activation of the mineralocorticoid receptor (MR) drives inflammation, fibrosis, and oxidative stress across tissues and organs, representing a fundamental mechanism underlying systemic metabolic disorders, diabetes, cardiovascular diseases, and kidney pathologies. Emerging clinical evidence indicates that the non‐steroidal mineralocorticoid receptor antagonist (ns‐MRA) finerenone can block MR with high selectivity, significantly reducing cardiorenal event risks in older patients and thereby enhancing overall health status. This offers a novel therapeutic avenue for the long‐term management of older patients burdened with multiple chronic comorbidities.

## Graphical abstract

Mineralocorticoid receptor (MR) overactivation drives inflammation, oxidative stress, and fibrosis in the heart, kidneys, and vasculature, leading to cardiorenal dysfunction. MR signaling promotes hypertrophy, remodeling, and injury through pathways like oxidative stress and inflammation, resulting in vascular stiffness and progressive organ damage. Finerenone, a selective non‐steroidal MR antagonist, effectively blocks MR overactivation, attenuating these pathological processes and improving cardiac and renal outcomes in elderly patients with multimorbidity.

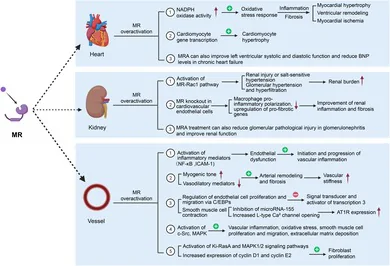

## 1 Introduction

With the sustained intensification of population aging in China, geriatric diseases are characterized by complexity, such as multimorbidity and multi‐system interactions, which pose new challenges for comprehensive management in geriatric medicine. Epidemiological studies show that approximately 40% of patients with chronic kidney disease (CKD) also have heart failure (HF), and the comorbidity rate of HF and CKD in older adults is as high as 30% to 50%. Notably, among older patients with CKD, approximately 60% of cases involve diabetic kidney disease (DKD), a typical form of metabolic renal injury [1]. More importantly, there exists a complex bidirectional promoting relationship between cardiovascular risk factors such as diabetes and hypertension, and common geriatric conditions like cardiovascular disease and CKD. This interaction significantly exacerbates the risk of multi‐organ damage and adverse clinical outcomes.

Overactivation of the Mineralocorticoid Receptor (MR) is a key pathophysiological mechanism driving the progression of age‐related comorbidities. MR is widely distributed in multiple vital organ systems, including the kidneys, heart, vascular endothelium, central nervous system, and adipose tissue, as well as immune cells. Under physiological conditions, it regulates key physiological processes such as water and electrolyte balance, blood pressure homeostasis, inflammatory responses, and neuroendocrine function. However, under aging and pathological conditions, sustained aberrant MR activation triggers a series of pathological changes, including inflammatory cascades, oxidative stress imbalance, fibrosis, and endothelial dysfunction. Through interactions across the cardiorenal, metabolic, neural, and immune systems, these pathological effects collectively constitute the core node of the MR‐related pathological network in geriatric comorbidities [2].

Based on this mechanistic understanding, intervention strategies targeting MR are garnering increasing attention in the academic community. Among these, the novel non‐steroidal mineralocorticoid receptor antagonist (ns‐MRA) finerenone exhibits distinct pharmacological advantages: It possesses high receptor selectivity and optimized tissue permeability, while significantly reducing the risk of hyperkalemia commonly associated with traditional MRAs, enabling efficient blockade of MR overactivation. From a mechanistic perspective, finerenone antagonizes MR‐mediated inflammatory responses, fibrotic processes, and oxidative stress pathways through multiple avenues, achieving synergistic regulation of multi‐system pathological processes. Multiple large‐scale clinical trials, including FIDELIO‐DKD and FIGARO‐DKD, have confirmed that finerenone significantly reduces the risk of cardiorenal composite outcomes in older patients, offering a novel therapeutic option with both efficacy and safety benefits for the clinical management of geriatric comorbidities.

This consensus systematically summarizes the research evidence and clinical practice experience of finerenone in treating geriatric diseases, and thoroughly explores its clinical utility in the management of geriatric comorbidities and intervention in geriatric syndromes. It aims to provide clinicians with evidence‐based standardized diagnosis and therapeutic recommendations, optimize individualized treatment strategies for older patients, and thus effectively improve clinical outcomes and quality of life.

### 1.1 Consensus Development Process

1. Consensus Initiating Organizations and Expert Panel Members

This consensus was developed by a consensus working group jointly established by experts from diverse fields such as geriatrics, cardiovascular medicine, endocrinology, nephrology, and epidemiology. The consensus development process was initiated in September 2024 and finalized in September 2025.

2 Consensus Users and Target Population

The primary users of this consensus are healthcare professionals at all levels of medical institutions, with particular relevance to patients in need of comprehensive geriatric disease management.

3 Selection and Determination of Key Questions

The working group conducted a systematic search and review of relevant literature based on the epidemiological status of geriatric diseases in China and the latest evidence‐based data on novel non‐steroidal MRAs. Building on this foundation and incorporating findings from surveys and interviews with selected experts, key clinical questions were identified. Through online questionnaires, the importance of initially proposed clinical questions was assessed, and clinicians were invited to supplement important but unaddressed issues. This process determined the core issues to be addressed by the consensus. Following multiple rounds of expert consultation, discussion, and feedback, the overall framework of this consensus was finalized.

4 Evidence Search

The consensus development working group established a dedicated evidence search and evaluation team to conduct literature searches focusing on its key questions. The databases searched included PubMed, Embase, China National Knowledge Infrastructure (CNKI), and Wanfang Data Knowledge Service Platform. Additionally, the working group reviewed and referenced the latest international guidelines and consensus statements related to CKD, hypertension, heart failure, diabetes, and Cardiovascular‐Kidney‐Metabolic (CKM) syndrome. The literature search cut‐off date was August 26, 2025. This consensus has been registered on the International Practice Guideline Registration for Transparency, with the registration number PREPART‐2025CN954.

5 Formulation of Recommendations

Based on the domestic and international evidence provided by the evidence search and evaluation team, and considering the current clinical practice of comprehensive management for elderly patients with chronic diseases in China, the expert panel formulated recommendations for each key question via the consensus conference method. From September 2024 to September 2025, the expert panel conducted three rounds of consensus workshops. Following open discussions, feedback, and revisions, a broad final consensus was reached.

## 2 Characteristics of the Mineralocorticoid Receptor and Age‐Related Changes

### 2.1 Physiological Characteristics

MR belongs to the nuclear receptor superfamily of steroid hormone receptors. Its physiological ligands are aldosterone and cortisol; progesterone or androgens and their derivatives can also bind to MR [3]. MR is expressed in cardiomyocytes, vascular endothelial cells, smooth muscle cells, renal tubular epithelial cells, macrophages, and adipocytes, among others [3]. In the kidney, the aldosterone‐MR complex binds to hormone response elements, activating sodium channels in renal tubular epithelial cells and the Na^+^, K^+^‐ATPase on the basement membrane. This promotes Na^+^ and Cl^−^ reabsorption while excreting H^+^ and K^+^, thereby regulating extracellular fluid volume and maintaining electrolyte balance [4], playing an important physiological role.

### 2.2 Pathological Characteristics

MR is involved in pathological injury processes across multiple systems and organs throughout the body. MR overactivation promotes oxidative stress, induces endothelial dysfunction, and mediates inflammatory and fibrotic processes. Increased expression of various inflammatory factors promotes the differentiation of inflammatory cells (such as macrophages and T cells) toward pro‐inflammatory phenotypes, further promoting and maintaining a chronic inflammatory state, ultimately leading to target organ damage [4, 5, 6] (Figure 1).

**FIGURE 1:**
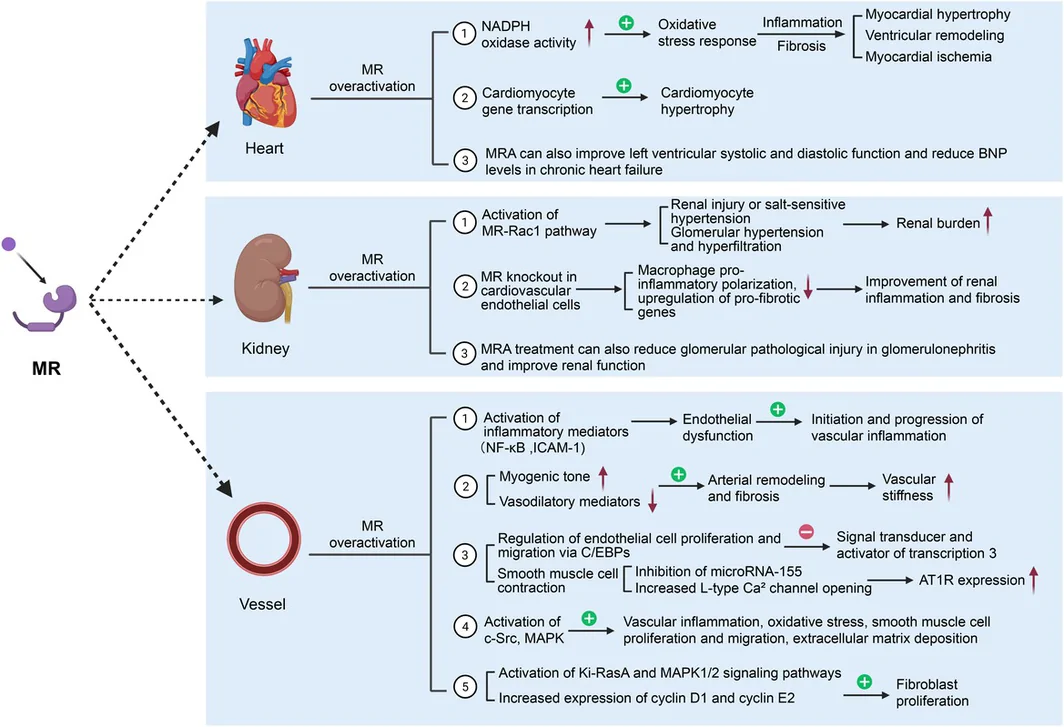
Tissue and Organ Distribution of Mineralocorticoid Receptors and the Mechanism.

MR overactivation in myocardial tissue increases NADPH oxidase activity, inducing a series of oxidative stress responses that mediate inflammatory and fibrotic processes, leading to the development and progression of myocardial hypertrophy, ventricular remodeling, and myocardial ischemia [7]. MR overactivation can also directly affect cardiomyocyte gene transcription, resulting in cardiomyocyte hypertrophy [8], suggesting that MR blockade is beneficial for improving cardiac remodeling [8, 9, 10]. In addition, MRA can improve left ventricular systolic and diastolic function and reduce plasma B‐type natriuretic peptide (BNP) levels in chronic heart failure [11, 12].

Renal MR overactivation can directly lead to renal injury or salt‐sensitive hypertension through the MR‐Rac1 pathway [13], or cause glomerular hypertension and hyperfiltration, increasing renal burden [14]. MR can additionally promote macrophage polarization toward a pro‐inflammatory phenotype and upregulate the expression of pro‐fibrotic genes, leading to subsequent renal inflammation and fibrosis [15]. MRA treatment can improve renal function [16].

Vascular MR activation can trigger the expression of inflammatory mediators (including nuclear factor‐κB and leukocyte adhesion molecules such as ICAM‐1), leading to endothelial dysfunction and promoting the initiation and progression of vascular inflammation [17, 18, 19]. MR activation can also promote arterial remodeling and fibrosis by increasing myogenic tone, inhibiting vasodilatory mediators, and other mechanisms, resulting in elevated vascular stiffness [20, 21]. In fibroblasts, MR activation promotes fibroblast proliferation by activating Ki‐RasA and mitogen‐activated protein kinase 1/2 (MAPK1/2) signaling and increasing the expression of cyclin D1 and cyclin E2 [22].

In addition, blocking MR can alleviate neuroinflammatory responses in the spinal cord of mice with experimental autoimmune encephalomyelitis [23]. In contrast, the MR agonist aldosterone can promote the proliferation of hippocampal neural stem cells and improve cognitive dysfunction in aged mouse models of cognitive impairment [24]. MR can also mitigate femoral head endothelial cell injury through central nervous system regulation. When hypothalamic paraventricular nucleus MR activity is inhibited by glucocorticoids (GCs), reduced sympathetic outflow leads to downregulation of Adrb2 receptors in femoral head endothelial cells—decreased expression of the glycolytic key enzyme PFKFB3—impaired angiogenesis. Central delivery of an MR agonist can reverse this process by restoring the sympathetic‐vascular signaling axis, thereby antagonizing the pathological progression of GC‐induced osteonecrosis [25].

### 2.3 Pharmacological Characteristics

Based on molecular structure, MRAs can be classified into steroidal and non‐steroidal types. Spironolactone is a steroidal MRA and the longest‐established in clinical practice; eplerenone is a highly selective steroidal MRA; finerenone is a highly selective non‐steroidal MRA launched in China in June 2022. For a comparison of their molecular structures and pharmacological properties, see Table 1.

1. **Steroidal MRAs:** Steroidal MRAs mainly include spironolactone and eplerenone. Eplerenone was derived via structural modification of aldosterone; both share a flat chemical steroidal backbone. The steroidal structure of spironolactone binds to the MR ligand‐binding domain, exerting an antagonistic effect on MR [26], and has strong antagonistic potency against MR. However, spironolactone has poor receptor selectivity and can also cross‐bind to androgen receptors and progesterone receptors, thus resulting in sex hormone‐related adverse reactions, most commonly gynecomastia in men [27]. Eplerenone features an optimized steroidal structure that reduces its affinity for androgen and progesterone receptors and increases its selectivity for the MR, but its antagonistic potency against the MR is only 1/40 that of spironolactone [26].
2. **Non‐steroidal MRAs:** Unlike steroidal MRAs, non‐steroidal MRAs are naphthyridine derivatives developed through high‐throughput screening of millions of compounds based on the dihydropyridine (DHP) scaffold. They possess a three‐dimensional structure with side chains, enabling more complete binding to the MR and greater antagonistic potency [26, 28]; moreover, they exhibit higher selectivity for MR with minimal affinity for androgen and progesterone receptors, thereby avoiding sex hormone‐related adverse effects [29].

**TABLE 1:** Comparison of three MRAs.

Classification	Steroidal MRA		Non‐steroidal MRA
	Spironolactone	Eplerenone	Finerenone
Molecular Structure	C_24_H_32_O_4_S	C_24_H_3_O_6_	C_21_H_22_N_4_O_3_
Structural Characteristics	Planar	Planar	Bulky
MR Antagonistic Potency	+++	+	+++
Selectivity for MR	+	+++	+++
Tissue Distribution	Kidney > Heart (approx. 6:1)	Kidney > Heart (approx. 3:1)	Balanced Distribution (1:1)
Oral Bioavailability	80%~90%	69%	86.50%
Protein Binding Rate	88%	33%~60%	92%
Metabolites	Multiple Active Metabolites (duration > 3 weeks)	Inactive Metabolites	Inactive Metabolites
Half‐life	> 20 h^a^	4~6 h^a^	2~3 h^b^
Common Adverse Reactions			
Sex Hormone‐related Adverse Reactions	++	+	—
Hyperkalemia	Yes	Yes	Uncommon

*Note:* MRA: Mineralocorticoid receptor antagonist; MR: Mineralocorticoid receptor.

^a^ In patients with heart failure.

^b^ In healthy subjects.

### 2.4 Age‐Related Changes in MR

Under physiological conditions, plasma renin activity decreases with age, attributable to age‐related renal changes (glomerulosclerosis, reduced number of functional nephrons) [30]. Meanwhile, the activity of the renin‐angiotensin‐aldosterone system (RAAS) is influenced by multiple integrated factors. Histopathological findings show persistent expression of aldosterone synthase in the adrenal zona glomerulosa of young individuals, whereas elderly adrenals exhibit reduced aldosterone synthase expression and more clusters of aldosterone‐producing cells. With increasing age, autonomous aldosterone secretion increases while physiological aldosterone secretion decreases [31, 32, 33]. Consequently, RAAS activation during aging can lead to cardiovascular disease [34]. Compared with younger patients, older patients with heart failure due to chronic ischemic heart disease exhibit a higher degree of RAAS activation [35]. Increased MR expression may drive age‐related cardiac dysfunction by exacerbating mitochondrial damage, increasing reactive oxygen species (ROS) accumulation, and inducing redox imbalance [36]. Additionally, aldosterone binding to MR and the angiotensin II type 1 receptor (AT1) activates oxidative stress pathways (Ras/NF‐κB, AP‐1/p53/p21), synergistically promoting vascular aging [37].

## 3 Evidence and Application Recommendations for Finerenone in Different Diseases in Older Adults

### 3.1 Chronic Kidney Disease in Older Adults

Two global multicenter phase III RCTs (FIDELIO‐DKD and FIGARO‐DKD) were designed to evaluate the efficacy and safety of finerenone in patients with type 2 diabetes mellitus (T2DM) and CKD. The FIDELIO‐DKD [38] study enrolled 5734 adult patients with T2DM and CKD who were randomized 1:1 to receive finerenone or placebo in addition to conventional therapy. Inclusion Criteria: Urinary albumin‐to‐creatinine ratio (UACR) ≥ 30 mg/g and < 300 mg/g with estimated glomerular filtration rate (eGFR) ≥ 25 mL/min/1.73 m^2^ and < 60 mL/min/1.73 m^2^, accompanied by diabetic retinopathy; or UACR ≥ 300 mg/g and ≤ 5,000 mg/g with eGFR ≥ 25 mL/min/1.73 m^2^ and < 75 mL/min/1.73 m^2^. The mean age of enrolled patients was 65.6 years, with a median follow‐up of 2.6 years. Results showed that, compared with the placebo group, finerenone significantly reduced the risk of the primary renal composite endpoint (kidney failure, sustained eGFR decline of ≥ 40% for over 4 weeks, or renal death) by 18%, reduced UACR by 31% after 4 months of treatment, and also reduced the risk of the primary cardiovascular composite endpoint (cardiovascular death, nonfatal myocardial infarction, nonfatal stroke, or hospitalization for heart failure) by 14%. The FIGARO‐DKD study [39] enrolled 7437 adult patients with T2DM complicated by CKD, randomized using the same method. Inclusion criteria were UACR ≥ 30 mg/g and < 300 mg/g with eGFR ≥ 25 mL/min/1.73 m^2^ and ≤ 90 mL/min/1.73 m^2^; or UACR ≥ 300 mg/g and ≤ 5,000 mg/g with eGFR ≥ 60 mL/min/1.73 m^2^. The mean age of enrolled patients was 64.1 years, with a median follow‐up of 3.4 years. Results showed that finerenone reduced the risk of the primary cardiovascular composite endpoint events by 13%, the risk of the renal composite endpoint by 23%, and UACR by 32% after 4 months of treatment. The FIGARO‐DKD study mainly enrolled patients with CKD stages 1–2 (61.7%), while the FIDELIO‐DKD study primarily included patients with CKD stages 3–4 (88.4%). Subgroup analyses from these two studies found no significant difference in the incidence of cardiorenal endpoint events between patients aged ≥ 65 years and those < 65 years [40].

The FIDELITY study [40] enrolled 697 patients in the Chinese subgroup. Results showed that finerenone reduced the risk of renal composite events by 43% in Chinese patients with T2DM‐associated CKD, and the cardiovascular composite outcome benefit was consistent with the global population. Subgroup analysis showed that in patients with comorbid atherosclerotic cardiovascular disease (ASCVD), the risk of cardiovascular composite endpoint events was reduced by 17%, while in patients without a history of ASCVD, it was reduced by 9%. Finerenone also significantly reduced the risk of the composite event of cardiovascular death or hospitalization for heart failure by 18% and all‐cause mortality by 15% in patients with a history of ASCVD [41]. The FIDELIO‐DKD and FIGARO‐DKD studies demonstrated that finerenone reduced the risk of new‐onset atrial fibrillation by 29% and the risk of new‐onset heart failure by 32% in patients with diabetes and CKD. In the FIDELIO‐DKD study, 8.1% (461 patients) had a history of atrial fibrillation. Subgroup analysis found that regardless of atrial fibrillation history, patients' cardiorenal benefits were consistent. In this study, 7.7% (436 patients) of enrolled patients had a history of heart failure, with 7.8% (571 patients) in the FIGARO‐DKD study. Subgroup analysis confirmed consistent patient benefits regarding renal, cardiovascular, and heart failure hospitalization‐related risks, irrespective of heart failure history [42, 43]. Therefore, for CKD patients at risk of ASCVD or with heart failure, priority should be given to finerenone. A large phase III clinical trial (FIND‐CKD study) investigating finerenone in non‐diabetic CKD (ndCKD) patients is currently underway. The study enrolled 1584 patients from 24 countries, who were randomly assigned to either the finerenone group or the placebo group. The median baseline eGFR and UACR were 46.7 mL/min/1.73m^2^ and 818.9 mg/g [44], respectively, and the results are expected to be published in 2026. Real‐world studies have shown that finerenone significantly reduces urinary protein levels and improves renal function in ndCKD patients, with good tolerability. A Chinese study involving 16 ndCKD patients with a mean age of 55.38 ± 14.37 years, baseline eGFR of 80.16 ± 31.46 mL/min/1.73 m^2^, and median UACR of 643.58 mg/g showed that after 3 months of combined finerenone treatment, UACR decreased by 44.52% compared to baseline [45]. Another Chinese study involving 37 ndCKD patients reached a similar conclusion, showing a median UACR reduction of 664.95 mg/g from baseline after 12 months of finerenone treatment [46]. Furthermore, a study of 36 patients with primary IgAN showed [47] that proteinuria levels were significantly reduced after 3 months of finerenone treatment. Another study involving 49 IgAN patients [48] reported a 24.86% reduction in UACR after 1 month of finerenone treatment, a 42.29% reduction after 3 months, and UACR levels continued to decrease after 6 months. In these studies, no significant decline in eGFR was observed in patients, and no drug discontinuations or hospitalizations due to hyperkalemia were reported [49]. Given the limited sample sizes in these studies, larger prospective clinical trials are warranted to confirm the relevant findings.

Renin‐angiotensin system inhibitors (RASi) are cornerstone medications for CKD treatment. Both the FIDELIO‐DKD and FIGARO‐DKD studies confirmed that adding finerenone to the maximally tolerated dose of a RASi can further improve cardiorenal outcomes in patients with CKD and T2DM. The 2024 KDIGO guidelines recommend that finerenone be co‐administered with a RASi in such patients (eGFR > 25 mL/min/1.73m^2^, normal serum potassium, and albuminuria); for patients unable to use a RASi due to blood pressure intolerance or other drug‐related adverse reactions, finerenone monotherapy is recommended to slow CKD progression and reduce the risk of adverse cardiovascular events. Sodium‐glucose cotransporter 2 inhibitors (SGLT2i), recommended as first‐line agents in CKD guidelines, significantly reduce the risk of kidney failure and cardiovascular events. The FIDELITY study [39] found that finerenone reduced the risk of cardiorenal composite endpoint events independently of SGLT2i use, but additional benefit could still be obtained when an SGLT2i was co‐administered with finerenone. Furthermore, the study found that co‐administration of an SGLT2i reduced the risk of finerenone‐related hyperkalemia and decreased the incidence of acute kidney injury (AKI). Real‐world data show that the triple regimen of RASi + SGLT2i + finerenone reduces UACR by 73%. This combination regimen demonstrates both efficacy and safety [50]. The recently published CONFIDENCE study, the first randomized controlled, double‐blind, double‐dummy, international multicenter study evaluating the simultaneous initiation of finerenone and an SGLT2i, demonstrated that in patients with T2DM‐related CKD, simultaneous initiation of combination therapy with finerenone and an SGLT2i significantly reduced urinary albumin‐to‐creatinine ratio (UACR) levels compared to empagliflozin or finerenone monotherapy. A rapid UACR reduction of up to 30% was observed as early as 14 days after treatment initiation, with a significant 52% reduction at 6 months, along with good safety and tolerability [51]. Given that UACR is an important mediator of cardiorenal outcomes, these data provide critical evidence for the clinical initiation of combination therapy.

Recommended Opinions:

1. Finerenone is recommended for older patients with CKD, eGFR ≥ 25 mL/min/1.73 m^2^, and albuminuria, regardless of diabetes status.
2. Finerenone is preferentially recommended in the following high‐risk populations: Patients at high risk of CKD progression, patients at high cardiovascular risk, patients with established atherosclerotic cardiovascular disease (ASCVD), and older patients with CKD and diabetes who have heart failure, irrespective of ejection fraction.
3. Finerenone is recommended in older patients with CKD and diabetes.
4. For older patients with CKD and diabetes, finerenone may be co‐administered with RASi and SGLT2i, or initiated simultaneously with SGLT2i as combination therapy.
5. In older CKD patients intolerant to RASi, finerenone monotherapy may be considered.

### 3.2 Heart Failure in Older Patients

1. Heart Failure With Reduced Ejection Fraction (HFrEF) in Older Patients

The ARTS trial showed that finerenone reduced BNP and NT‐proBNP levels in patients with chronic HFrEF and CKD comparable to that of spironolactone, with a significantly smaller impact on eGFR than spironolactone [52]. The ARTS‐HF trial showed that in patients with chronic HFrEF and T2DM and/or CKD, the proportion of patients achieving a > 30% reduction in NT‐proBNP levels was similar between the finerenone group and the eplerenone group, but the incidence of composite endpoint events was significantly lower in the finerenone group [53]. Compared to spironolactone and eplerenone, finerenone exerts a smaller effect on serum potassium. Based on the RALES and EMPHASIS‐HF studies, which demonstrated that steroidal mineralocorticoid receptor antagonists (MRAs) reduce cardiovascular events in chronic HFrEF, recent European and American heart failure guidelines as well as Chinese heart failure guidelines recommend the use of spironolactone or eplerenone for HFrEF. Although the ARTS and ARTS‐HF trials showed that finerenone is non‐inferior to steroidal MRAs in treating chronic HFrEF and CKD, it currently has no approved indication for HFrEF treatment. It may be considered for use based on clinical circumstances, such as in patients with chronic HFrEF and CKD.

Recommended Opinions:

1. Older patients with HFrEF and comorbid CKD may be considered for finerenone treatment.
2. For older patients with HFrEF who are intolerant to sMRA, the ns‐MRA finerenone may be considered.

1. Heart Failure With Mildly Reduced Ejection Fraction/Heart Failure With Preserved Ejection Fraction (HFmrEF/HFpEF) in Older Patients. [Correction added on 9 September 2026 after first online publication: The section heading “Heart Failure With Mildly Reduced Ejection Fraction/Heart Failure With Preserved Ejection Fraction (HFmrEF/HFpEF) in Older Patients” has been moved under Section 3.2, “Heart Failure in Older Patients,” as the second subsection.]

FINEARTS‐HF, the first study to explore the efficacy and safety of a non‐steroidal MRA in patients with HFmrEF/HFpEF, enrolled 6001 symptomatic heart failure patients with left ventricular ejection fraction (LVEF) ≥ 40% from 37 countries/regions worldwide. Patients were randomized 1:1 to receive either finerenone (20 mg or 40 mg once daily) or placebo. Regarding efficacy, over a median follow‐up of 32 months, finerenone reduced the risk of the primary endpoint by 16% and the overall risk of heart failure events by 18% compared with placebo in patients with HFmrEF/HFpEF. On day 28 of finerenone treatment, the risk of cardiovascular death and overall heart failure events decreased by 38%. Improvement in the Kansas City Cardiomyopathy Questionnaire Total Symptom Score (KCCQ‐TSS) was significantly greater in the finerenone group than in the placebo group. Regarding safety, the incidence of serious adverse events was similar between the finerenone and placebo groups (38.7% vs. 40.5%). Finerenone increased the risk of hyperkalemia (9.7% vs. 4.2%), but the overall risk of hospitalization and treatment discontinuation due to hyperkalemia was low; it also reduced the risk of hypokalemia (4.4% vs. 9.7%) [54]. Regarding other endpoint events: Finerenone reduces the risk of new‐onset diabetes [55]; leads to early and sustained reduction in proteinuria and reduces the risk of new‐onset microalbuminuria and macroalbuminuria [56]; and although it increases the risk of hyperkalemia and reduces the risk of hypokalemia, after dose adjustment, patients who develop hyperkalemia still benefit [57]. A secondary analysis of patients with different baseline characteristics found that: Finerenone demonstrated consistent benefit in the primary endpoint across sex [58], age [59], LVEF (< 50%, ≥ 50% to < 60%, ≥ 60%) [60], SGLT2i use [61], and patients with recent heart failure exacerbations [62]. Finerenone reduced cardiovascular death and overall heart failure events across all age groups (40–66 years, 67–73 years, 74–79 years, ≥ 80 years), with no age‐related differences in hypotension, creatinine elevation, hyperkalemia, or hypokalemia [63]. FINE‐HEART further pooled data from the FIDELIO‐DKD, FIGARO‐DKD, and FINEARTS‐HF studies and found that, compared with placebo, finerenone reduced the composite risk of cardiovascular death or heart failure hospitalization and the risk of new‐onset atrial fibrillation in patients with HFmrEF/HFpEF, with no increase in serious adverse events [64].

Recommended Opinions:

1. Older patients with HFmrEF/HFpEF should receive finerenone as early as possible to reduce the risk of cardiovascular death or worsening HF events.
2. The benefit of finerenone in older patients with HFmrEF/HFpEF showed no significant differences regardless of sex, LVEF, BMI, SGLT2i use, or recent heart failure exacerbations.

### 3.3 Hypertension in Older Patients

Increased arterial stiffness is the primary pathological basis of isolated systolic hypertension in older patients. MR activation in vascular smooth muscle cells mediates aging‐related arterial stiffness, and administration of spironolactone or MR knockout in smooth muscle cells inhibits aging‐related arterial stiffness in mice [65, 66]. Advanced age, diabetes, and impaired renal function are important risk factors for salt‐sensitive hypertension, and MR activation plays a crucial role in the development of salt‐sensitive hypertension and associated cardiorenal damage [67]. Blocking MR overactivation reduces blood pressure, effectively protects cardiorenal function, and improves vascular remodeling.

Traditional MRAs spironolactone and eplerenone can be used to treat resistant hypertension, but clinical trial evidence supporting the use of the novel non‐steroidal MRA finerenone for the treatment of primary hypertension is currently lacking. In the FIDELITY study [40] (a pooled analysis of two RCTs of finerenone in diabetic kidney disease, the FIDELIO‐DKD^38^ and FIGARO‐DKD^39^ studies), with well‐controlled baseline blood pressure (baseline systolic blood pressure 136.7 mmHg), the finerenone group reduced systolic blood pressure by 3.7 mmHg compared with the placebo group. An indirect comparative analysis between the resistant hypertension subgroup in the FIDELITY study and the AMBER study (which used spironolactone to treat patients with resistant hypertension and chronic kidney disease) found that in patients with resistant hypertension and CKD, finerenone was associated with a smaller reduction in systolic blood pressure, a lower risk of hyperkalemia, and a lower risk of treatment discontinuation compared with spironolactone [13].

The ARTS‐HF study compared the efficacy of eplerenone and finerenone in patients with worsening chronic heart failure and diabetes and/or chronic kidney disease, finding no significant difference in their effects on systolic blood pressure, with a mean reduction of less than 3 mmHg.

A post hoc analysis of the ambulatory blood pressure monitoring subgroup from the ARTS‐DN study (an RCT of finerenone in diabetic kidney disease) [68], which included 240 patients with ABPM records (mean age 63.9 ± 9.4 years), showed that compared with placebo (baseline systolic blood pressure 138 mmHg), finerenone 10 mg, 15 mg, or 20 mg/day for 90 days added to angiotensin‐converting enzyme inhibitor (ACEI) or angiotensin receptor blocker (ARB) therapy reduced mean office systolic blood pressure by 1.4 mmHg, 6.5 mmHg, and 3.6 mmHg, respectively. Office blood pressure measurements showed high variability and poor consistency. The ambulatory blood pressure monitoring results indicated that compared with placebo, finerenone 10 mg, 15 mg, and 20 mg/day reduced 24‐h mean systolic blood pressure by 8.3 mmHg, 11.2 mmHg, and 9.9 mmHg, respectively. These results were not influenced by age group (> 65 years vs. ≤ 65 years), suggesting that ABPM provides a more accurate assessment of the antihypertensive effect of finerenone. Despite its relatively short half‐life, finerenone provides sustained blood pressure reduction throughout both day and night.

In the FINEARTS‐HF study [54] (an RCT of finerenone in heart failure with preserved or mildly reduced ejection fraction), the 6001 enrolled patients had a mean age of approximately 72 years, with about 89% having hypertension and a mean baseline systolic blood pressure of approximately 130 ± 15 mmHg. When the maximum dose of finerenone (20–40 mg) was added to background therapy including β‐blockers, ACEIs/ARBs/ARNIs, calcium channel blockers, and diuretics for 6 months, finerenone reduced mean systolic blood pressure by 3.4 mmHg compared to the control group.

MRAs are the optimal pharmacological treatment for primary aldosteronism (PA), and spironolactone is the first‐line therapy for PA. Evidence for finerenone in treating PA is limited. A recent study, the first to evaluate the efficacy and safety of finerenone in PA patients, showed that finerenone (20–40 mg/day) was as effective as spironolactone (20–40 mg/day) in lowering blood pressure after 8 weeks of treatment and was associated with a better safety profile [69].

Recommended Opinions:

1. Finerenone is recommended for patients with primary hypertension and comorbid diabetic kidney disease.
2. Finerenone is recommended for patients with primary hypertension and comorbid heart failure with preserved or mildly reduced ejection fraction.
3. Finerenone may be considered for older hypertensive patients who are intolerant to spironolactone due to adverse effects, particularly in those with isolated systolic hypertension and increased arterial stiffness, or in those with salt‐sensitive hypertension.
4. ABPM is recommended for assessing the antihypertensive effect of finerenone.

## 4 Recommendations for the Use of Finerenone in Common Clinical Scenarios in Older Patients

1. Age and Drug Dosage: In the FIDELIO‐DKD study, the mean age of patients in the finerenone group was 65.4 ± 8.9 years. Subgroup analysis found no overall differences in safety or efficacy between patients aged ≥ 65 years and younger patients, indicating that no dose adjustment is necessary based on age [70]. In the FIGARO‐DKD study, the mean age of patients in the finerenone group was 64.1 ± 9.7 years. Therefore, the recommended dosage for non‐oldest‐old patients can follow that for non‐older adults, while dosage recommendations for the oldest‐old population require further research and data summarization. In clinical practice, the initial dose can be appropriately adjusted and dynamically tailored based on individual patient assessment. Drug Interactions: Co‐administration with strong CYP3A4 inhibitors (e.g., ketoconazole) should be avoided to prevent increased plasma concentrations; when co‐administered with ACEIs/ARBs, a lower starting dose is required.
2. Medication Use in Multimorbidity: The prevalence of multimorbidity is extremely high among older patients with conditions such as diabetes, chronic kidney disease (CKD), and chronic heart failure. Studies in China have found that it is very common for patients with type 2 diabetes (T2DM) to have coexisting cardiorenal diseases or metabolic abnormalities (such as hypertension and obesity) [71]. Multimorbidity is also extremely prevalent in patients with CKD and cardiac dysfunction, especially among older patients. In the context of multimorbidity, the use of finerenone requires stricter adherence to the principles of indication and dose adjustment. Closely monitor blood pressure, serum potassium, and renal function. Exercise caution when co‐administering with other high‐risk medications (such as RAAS inhibitors) and carefully assess patient tolerance to achieve precise intervention that blocks the common pathway of MR overactivation. Comprehensively evaluate changes in the patient's eGFR, serum potassium levels, and blood pressure before and after finerenone use, and develop personalized treatment plans based on the risks and benefits for each individual patient. Based on the characteristics of geriatric medicine, promptly initiate a multidisciplinary team and working mechanism to ensure medication safety and efficacy in older patients with multiple comorbidities.
3. Effects of Finerenone on Cognitive Function: MR is abundantly expressed in the brain (particularly in the hippocampus and prefrontal cortex), is associated with the regulation of salt intake and psychological stress responses [72], and is involved in modulating stress responses, inflammation, oxidative stress, and neuronal plasticity. Overactivation of MR may be associated with neurodegeneration and cognitive dysfunction. A meta‐analysis showed that the MR antagonist spironolactone exhibited mixed effects on cognitive function: In healthy subjects, it improved spatial memory under stress and prevented stress‐induced suppression of medial temporal lobe activity, but impaired working memory and selective attention. In patients with psychiatric disorders, spironolactone alleviated cognitive empathy deficits in patients with major depressive disorder and improved working memory in patients with type I bipolar disorder. In patients with cardiovascular disease, spironolactone improved cognitive scores and hippocampal memory function, but had no effect on non‐hippocampal memory [73]. In an angiotensin II‐induced hypertensive mouse model, the MR antagonist eplerenone has been shown to improve cerebrovascular function and cognitive function in hypertensive patients [74]. Direct effects of finerenone on cognitive function have not been reported; potential impacts can only be analyzed based on its pharmacological mechanisms and existing data. By inhibiting MR overactivation, finerenone may reduce neuroinflammation and oxidative stress, theoretically providing protective effects on cognitive function (especially in patients with diabetes or hypertension, as these conditions themselves increase the risk of cognitive impairment). Some data suggested that finerenone can improve vascular endothelial function and reduce systemic inflammation, potentially indirectly slowing the decline of central nervous system function. Finerenone has low lipophilicity and limited permeability across the blood–brain barrier, making direct effects on cognition less likely. However, by improving glycemic control, blood pressure, and renal function, finerenone may potentially reduce the risk of vascular dementia or Alzheimer's disease in older patients. In reports of completed clinical trials, no significant signals of cognitive impairment (such as memory decline or attention deficits) directly attributable to finerenone have been identified. Certainly, prolonged hyperkalemia can indirectly affect cognitive function.
4. Effects of Finerenone on Older Malnourished Patients: There is limited research on the association between finerenone and malnutrition in older adults; potential impacts and considerations can be analyzed from several aspects. First, many older adults often experience malnutrition due to chronic diseases, medication side effects, or physiological decline, which can lead to muscle atrophy, decreased renal function, or a hypoaldosteronism state, making them more susceptible to electrolyte disturbances (such as hyperkalemia). Older malnourished patients may have hypoalbuminemia, which affects drug protein binding. Finerenone has a high protein binding rate (approximately 92%) [75, 76]. Hypoalbuminemia may increase free drug concentrations, necessitating monitoring for adverse effects (such as decreased blood pressure and changes in renal function). Therefore, finerenone should be used with caution in patients with severe malnutrition (e.g., BMI < 18.5) who are at risk of hyperkalemia. When clinical use of this drug is necessary, consultation with a dietitian is recommended to collaboratively optimize the patient's diet (e.g., controlling high‐potassium food intake).
5. Effects of Finerenone in Older Frail Patients: Although Phase III clinical trials (FIDELIO‐DKD, FIGARO‐DKD) included a high proportion of older patients (approximately 40% were ≥ 65 years old), there have been few subgroup analyses specifically focusing on frail older patients [38, 39]. Currently, research and clinical data on finerenone and frailty in older patients are limited. Since finerenone may cause hyperkalemia (particularly in older patients with renal insufficiency), leading to muscle weakness, arrhythmia, and even an increased risk of falls, this may indirectly aggravate the development and progression of frailty. Additionally, this drug may cause a reduction in blood pressure; if older patients have hypovolemia or orthostatic hypotension, it may further increase the risk of dizziness and falls. Furthermore, finerenone may delay the progression of kidney disease through its anti‐inflammatory and anti‐fibrotic effects [77], while reducing recurrent hospitalizations in patients with chronic heart failure [78], potentially indirectly preventing and improving frailty.
6. Rational Use of Comprehensive Geriatric Assessment (CGA): CGA is a multidimensional interdisciplinary assessment designed to identify the medical, psychological, functional, and social needs of older patients to develop individualized intervention plans. Older patients may undergo CGA based on their clinical circumstances, such as for finerenone indication screening. During polypharmacy, CGA can also be used to assess the risk of drug interactions [79], such as risk avoidance when finerenone is co‐administered with strong CYP3A4 inhibitors. If co‐administered with ACEIs/ARBs, potassium supplements, or potassium‐sparing diuretics, close monitoring of serum potassium is recommended. CGA can assess the physical function and cognitive status of older patients, including monitoring medication adherence, and propose simplified medication regimens or caregiver‐assisted management based on the assessment results. Assessment of fall risk in older patients can reduce the increased risk of falls due to hyperkalemia‐induced muscle weakness or arrhythmia. In daily clinical practice, baseline assessment is recommended for selected patients, and the decision to initiate finerenone should be made after a comprehensive CGA.

Recommended Opinions:

1. No dosage adjustment is required when administering finerenone to older patients; however, clinical evidence remains limited for the very elderly. Careful patient assessment before treatment initiation and close monitoring after administration are essential to ensure therapeutic safety.
2. In older patients with multimorbidity, individualized treatment strategies and multidisciplinary collaboration are essential to minimize the potential risks of finerenone while maximizing its cardiorenal protective effects.
3. There is currently no evidence that finerenone impairs cognitive function in older patients, and its theoretical neuroprotective effects require further verification.
4. Malnourished older patients, particularly those with hypoalbuminemia, are at further increased risk of hyperkalemia when treated with finerenone. A careful assessment of the benefit–risk balance is essential, and multidisciplinary collaborative management should be implemented when necessary.
5. In older patients, finerenone may indirectly ameliorate frailty through its cardiorenal protective effects; however, clinicians should remain vigilant for potential electrolyte disturbances and worsening renal function. For patients with existing frailty or those at high risk of frailty, collaborative care involving geriatrics or a multidisciplinary team is recommended.
6. The decision to use finerenone should be guided by the patient's individual conditions combined with CGA results, with particular attention to renal function, electrolytes, polypharmacy, and patients' physical and cognitive status, ensuring an optimal balance between cardiorenal protection and medication safety.

## 5 Summary and Outlook

A growing body of basic and clinical research indicates that MR overactivation plays a significant role in inflammatory fibrosis and dysfunction of the cardiovascular system, kidneys, and other organs. As a novel non‐steroidal mineralocorticoid receptor antagonist, finerenone has demonstrated clinical value in improving common geriatric diseases such as CKD, heart failure, and hypertension by effectively blocking the pathological signaling pathways induced by abnormal MR activation.

Currently, numerous ongoing clinical trials, both domestically and internationally, are dedicated to evaluating the value of finerenone in various cardiorenal diseases. In the field of heart failure, the REDEFINE‐HF, CONFIRMATION‐HF, and FINALITY‐HF studies will evaluate the efficacy and safety of finerenone in a broader spectrum of heart failure patients. In the field of nephrology, the FIND‐CKD study aims to evaluate the efficacy and safety of finerenone in non‐diabetic CKD patients. The FINE‐ONE study will explore the efficacy and safety of finerenone in patients with type 1 diabetes and comorbid CKD. Furthermore, the FINE‐REAL study will provide real‐world evidence for the use of finerenone in treating diabetes‐related CKD in routine clinical practice.

In summary, this consensus outlines the current application prospects of MR overactivation and the ns‐MRA finerenone in optimizing the comprehensive management of common geriatric diseases. Looking ahead, we anticipate that more high‐quality clinical data and basic research will continuously advance the refinement of finerenone treatment strategies and explore its broader application in the prevention and management of multi‐system diseases, aiming to provide older patients with safer, more effective, and personalized treatment options.

## Author Contributions

Initiated the organization of this consensus: Xiaoming Wang and Cuntai Zhang. Writing the initial draft (including substantive translation): Editing group of the Chinese Expert Consensus on the Clinical Application of Finerenone in Geriatric Comorbidities. Critical review and revision: Xiaoming Wang, Cuntai Zhang, Cardiovascular Group, Geriatrics Branch of Chinese Medical Association.

## Funding

This guideline was funded by the Key Research and Development Program of Shaanxi Province (2024SF‐ZDCYL‐01‐12). International Practice Guideline Registration for Transparency (PREPART‐2025CN954).

## Disclosure

Academic Consultants (in alphabetical order of surname) Xiaoying Li (The Second Medical Center, Chinese PLA General Hospital). Jianye Wang (Beijing Hospital, National Center of Gerontology). Huan Xi (Beijing Hospital, National Center of Gerontology). Pulin Yu (Beijing Hospital, National Center of Gerontology).Qiang Zhang (Tianjin Medical University General Hospital). Methodology Experts Lei Shang (Air Force Medical University). Bo Wang (Air Force Medical University). Writing Committee Experts (in alphabetical order of surname) Ming Cao (Tongji Hospital, Tongji Medical College, Huazhong University of Science and Technology). Qingli Cheng (The Second Medical Center, Chinese PLA General Hospital). Zhanyi Lin (Guangdong Provincial People's Hospital). Yang Sun (Xijing Hospital, Air Force Medical University). Wen Tian (The First Hospital of China Medical University). Xiaoming Wang (Xijing Hospital, Air Force Medical University). Jinhui Wu (West China Hospital, Sichuan University). Cuntai Zhang (Tongji Hospital, Tongji Medical College, Huazhong University of Science and Technology). Discussant Experts (in alphabetical order of surname). Jian Cao (Chinese PLA General Hospital). Long Chen (Fuwai Hospital, Chinese Academy of Medical Sciences·Shenzhen). Xujiao Chen (Zhejiang Hospital) Biao Cheng (Sichuan Provincial People's Hospital). Wei Cui (The First Affiliated Hospital of Xi’an Jiaotong University) Guoxian Ding (Jiangsu Province Hospital). Mingge Ding (The Second Affiliated Hospital of Xi’an Jiaotong University). Ying Ding (The Second Medical Center, Chinese PLA General Hospital). Jin Fan (Taikang Yanyuan Rehabilitation Hospital Beijing). Ningyuan Fang (Renji Hospital, Shanghai Jiao Tong University School of Medicine). Pan Gao (The First Hospital Affiliated to Army Medical University). Yifang Guo (Hebei Provincial People's Hospital). Lulu Han (Shengjing Hospital of China Medical University). Wen He (The First Affiliated Hospital, Sun Yat‐sen University). Huashan Hong (Fujian Medical University Union Hospital). Yu Hu (Zhongshan Hospital, Fudan University). Gairong Huang (Henan Provincial People's Hospital). Weiguo Jia (West China Hospital, Sichuan University). Xin Jiang(Shaanxi Provincial People's Hospital). Xin Jiang (Shenzhen People's Hospital). Rong Li (Xijing Hospital, Air Force Medical University). Yan Li (The First People Hospital Of Yunnan Province). Jiangjiu Liang (The First Hospital Affiliated with Shandong First Medical University). Chengyun Liu (Union Hospital, Tongji Medical College, Huazhong University of Science and Technology). Deping Liu (Beijing Hospital). Feng Liu (Guangzhou First People's Hospital). Yongming Liu (The First Hospital of Lanzhou University). Youshuo Liu (The Second Xiangya Hospital of Central South University). Ze Liu (General Hospital of Southern Theater Command). Da Liu (The First Affiliated Hospita of Shihezi University School of Medicine). Huiling Lou (Guangzhou First People's Hospital). Xiang Lu (Nanjing Medical University). Yongjun Mao (The Affiliated Hospital of Qingdao University). Xiaoxuan Ning (Xijing Hospital, Air Force Medical University). Boqing Ou (Hunan Provincial People's Hospital). Chengdong Qiao (The First Hospital of Lanzhou University). Wei Qiao (China‐Japan Friendship Hospital). Mingzhao Qin (Beijing Tongren Hospital, Capital Medical University). Mingzhi Shen (Hainan Hospital, Chinese PLA General Hospital). Lin Shen (Qilu Hospital of Shandong University). Kailei Shi (Huadong Hospital Affiliated to Fudan University). Hui Su (Xijing Hospital, Air Force Medical University). Xianming Su (The First Affiliated Hospital of Xi'an Jiaotong University). Jun Tao (The First Affiliated Hospital, Sun Yat‐sen University). Tao Tian (Linyi People's Hospital). Wen Tian (The First Hospital of China Medical University). Ling Tu (Tongji Hospital, Tongji Medical College, Huazhong University of Science and Technology). Ke Wan (West China Hospital, Sichuan University). Yuehui Wang (The first hospital of Jilin University). Zhaohui Wang (Union Hospital, Tongji Medical College, Huazhong University of Science and Technology). Hong Wang (First Affiliated Hospital of Xinjiang Medical University). Hong Wen (The First Affiliated Hospital of Guangxi Medical University). Zhenli Wu (Inner Mongolia People's Hospital). Jinhui Wu (West China Hospital, Sichuan University). Jing Wu (The Third People Hospital of Chengdu). Xiaohe Wu (Jiangxi Provincial People's Hospital). Ling Xi (First Hospital of Shanxi Medical University). Kun Xing (Shaanxi Provincial People's Hospital). Hong Xu (People's Hospital of Xinjiang Uygur Autonomous Region). Lin Xu (General Hospital of Southern Theater Command). Di Xu (Jiangsu Province Hospital). Guang Yang (The First Medical Center, Chinese PLA General Hospital). Li Yang (General Hospital of Ningxia Medical University). Li Yang (Yan'an Hospital of Kunming City). Ruiying Yang (General Hospital of Ningxia Medical University). Yunmei Yang (The First Affiliated Hospital Zhejiang University School of Medicine). Qin Zhang (The First Affiliated Hospital Zhejiang University School of Medicine). Min Zeng (Hainan Medical University). Zhiyu Zeng (Guangxi Medical University). Weihong Zhao (Jiangsu Province Hospital). Yingxin Zhao (Beijing Anzhen Hospital Capital Medical University). Xiaohui Zhou (The First Affiliated Hospital of Xinjiang Medical University). Pengli Zhu (Fujian Provincial Hospital). Aiqin Zhu (Qinghai Provincial People's Hospital). Xin Zhuge (Tianjin Medical University General Hospital). Writing Secretary: Liming Hou (Xijing Hospital, Air Force Medical University).

## Conflicts of Interest

The authors declare no conflicts of interest.

## Data Availability

This article is an expert consensus and a literature‐based review and does not involve original research data.
